# Evaluation of a Community Suicide Prevention Project (Roots of Hope): Protocol for an Implementation Science Study

**DOI:** 10.2196/39978

**Published:** 2023-06-14

**Authors:** Brian L Mishara, Anh Tu Tran, Lianna Chondo, Amanda Demmer, Laura Harris-Lane, Sheila Harper, Jalila Jbilou, Hazel Williams-Roberts, Tanya Wilson

**Affiliations:** 1 Centre for Research and Intervention on Suicide, Ethical Issues and End-of-Life Practices Université du Québec à Montréal Montréal, QC Canada; 2 Psychology Department Université du Québec à Montréal Montréal, QC Canada; 3 City of Edmonton Edmonton, AB Canada; 4 Waterloo Region Suicide Prevention Council Waterloo, ON Canada; 5 Memorial University of Newfoundland St. John's, NL Canada; 6 R.A. Malatest & Associates Ltd. Edmonton, AB Canada; 7 School of Psychology Faculty of Health Sciences and Community Service Université de Moncton Moncton, NB Canada; 8 Centre de formation médicale du Nouveau-Brunswick Faculty of Medicine Université de Sherbrooke Sherbrooke, QC Canada; 9 Saskatchewan Health Authority Saskatoon, SK Canada; 10 Horizon Health Network Fredericton, NB Canada

**Keywords:** suicide, prevention, community, evaluation, implementation science, implementation research, impact, mental health, psychological health, public health, community health

## Abstract

**Background:**

Roots of Hope (RoH) is a multisite Canadian community-based suicide prevention initiative developed by the Mental Health Commission of Canada (MHCC), which is based on evidence for intervention effectiveness and World Health Organization recommendations. Seven communities developed local activities in the following 5 pillars: specialized supports, training and networks, public awareness, means safety, and evaluation research.

**Objective:**

We aim to use an implementation research approach to understand the RoH model for reducing suicidal behaviors and their impacts in communities, and the lessons learned for the equitable development and implementation of RoH in different contexts. Moreover, we want to understand how the program is implemented in relation to the context, the causal pathways, and the factors influencing successful implementation. The evaluation includes assessments of short-term and intermediate effects at each site and overall.

**Methods:**

The principal investigator (PI) developed a consensus among local research coordinators on common approaches and indicators through ongoing participation in an online community of practice, and regular virtual and in-person meetings. At the completion of the pilot phase, the PI will summarize evaluation results across sites and conduct pooled analyses. The RoH theory of change and evaluation model shows how evaluation activities from the planning phase through the implementation of activities in each of the pillars can help clarify the viability of the RoH model and identify factors that facilitate and inhibit effective and equitable implementation in different contexts. Beginning with a situational analysis to identify resources in each community and local specificities, we will examine the implementation characteristics of conformity, dosage, coverage, quality, utility, equity, appreciation, facilitators, and impediments. Evaluation of short-term effects will focus on changes in knowledge, attitudes, behaviors, help-seeking, service use, stigma, media reports, empowerment, and care experiences. Intermediate effects, long-term effects, and impact will include assessments of the changes in suicides, suicide attempt rates, and suicide risk indicators. A variety of data sources, both quantitative and qualitative, will be used.

**Results:**

The quantitative and qualitative data from all sites will be summarized by the PI in March 2023 to draw conclusions to help the MHCC in its improvements to the RoH model, and to inform communities about how to better implement RoH. Since the COVID-19 pandemic occurred at the beginning of program implementation, its impact and influence will be documented. The validity of RoH in contributing to the prevention of suicides and suicidal behaviors will be clarified in a variety of contexts. The final evaluation report will be available in September 2023.

**Conclusions:**

The evaluation results, including the identification of factors that facilitate and inhibit the implementation of RoH and the adaptations to challenges, will be useful to the MHCC, current RoH communities, and those considering adopting the RoH model.

**International Registered Report Identifier (IRRID):**

DERR1-10.2196/39978

## Introduction

### Background

Roots of Hope (RoH) is a multisite national suicide prevention project initiated by the Mental Health Commission of Canada (MHCC) [1]. Its goal is to develop an evidence base, including best practices and suicide prevention guidelines and tools, to support the scaling up and implementation of a “made-in-Canada” community suicide prevention model across the country.

RoH was developed by the MHCC, based upon a systematic review the Commission conducted on the effectiveness of various suicide prevention programs and activities, as well as their review of evidence-based recommendations for community suicide prevention activities by the World Health Organization (WHO) [2,3]. The WHO proposes that key risk factors for suicide be aligned with relevant interventions at the societal, community, relationship, and individual levels. One of their key messages is that “Communities play a critical role in suicide prevention” [4]. This approach, which promotes community actions in suicide prevention, is mirrored in the comprehensive national Canadian Suicide Prevention Framework [5]. RoH provides structures around which communities can tailor their suicide prevention efforts to meet their unique needs. Each community develops strategies and activities in each of 5 “pillars” identified by the Mental Health Commission, which are evidence-based areas of prevention activities that can be found in all national suicide prevention programs that follow the WHO recommendations. The pillars are as follows:

1. Specialized supports: Prevention, crisis, and postcrisis services, such as peer support, support groups (including self-help), workplace interventions, and coordinated planning and access to services. 
2. Training and networks: Training and learning opportunities for health care providers, such as physicians and nurse practitioners, mental health workers, as well as community gatekeepers, such as first responders, human resource staff, and teachers. 
3. Public awareness campaigns: Locally driven campaigns to promote mental health awareness (posters, brochures, social media, etc) and collaboration with the media, with the goal of creating an effective suicide prevention awareness campaign and having safer conversations about suicide, including on social media. 
4. Means safety: Identification of the methods or places where a high number of suicides occur and implementation of measures to ensure safe access to them. For example, building barriers on bridges or railway crossings, protocol for medication access, etc. These include helping to reduce suicides by creating a safer home. 
5. Evaluation research: Setting research priorities, conducting surveillance, and monitoring and evaluating the implementation (using an implementation science approach) and effects of the project at each site and overall. 

### Evaluation Objectives

The overall objective of the evaluation is to understand RoH as a comprehensive model to reduce suicidal behaviors and their impacts in communities. Specific aspirational questions concerning this understanding that were formulated by the MHCC at the start of the project are included in Multimedia Appendix 1. An addition to these questions was made because of the timing of the implementation of the programs (an assessment of the impact of COVID-19 on the implementation and potential effects of the programs and their activities). The evaluation involves obtaining qualitative and quantitative data, including perspectives on the implementation and effects from members of the community, caregivers, and various stakeholders. The final report on the evaluation will identify lessons learned that could be useful for other communities implementing the model, and issues of equity and factors affecting the implementation will be discussed. In addition, actual and perceived impacts of the implementation of RoH will be assessed. We plan to analyze across sites the overall implementation and effects of common activities within each of the pillars, in order to better understand what appears to be effective and which factors influence the implementation of activities in different contexts. In addition to common quantitative metrics, a qualitative assessment will be carried out through semistructured interviews and focus groups. Analyses will include the assessment of possible unintended outcomes. In the RoH model, all of the areas of action have empirical evidence supporting the effectiveness of many activities. Most of the research has occurred in other cultures and in populations that may differ in terms of age, socioeconomic status, and local practices.

### Roles of the Principal Investigator and Local Research Teams, and Development of the Evaluation Plan 

In May 2018, Professor Brian L Mishara, Director of the Centre for Research and Intervention on Suicide, Ethical Issues and End-of-Life Practices (CRISE) and Psychology Professor at the Université du Québec à Montréal was appointed by the MHCC as principal investigator (PI) for the evaluation research of the RoH project. The PI (BLM) received funding for administrative support from the MHCC, which was used to hire an assistant to serve as research coordinator (ATT). The role of the PI is to develop a consensus on research methodologies and, to the extent possible, ensure that local research teams use the same common indicators in their evaluations. The consensus, which is reflected in this evaluation research plan, was developed over the course of 2 years through regular in-person and online meetings, and participation in a community of practice for only the research coordinators and a combined community of practice with the researchers and the implementation coordinators for each site. They also used a RoH research community of practice website to share information among local coordinators. Information about how to conduct the evaluation was discussed, modified, and approved by the lead researchers in each of the provinces and then by all the provincial intervention coordinators and the MHCC. Some of the tools and indicators that were adopted had been developed or used already in one or more communities as part of their ongoing evaluation activities. Some were created or adapted by the PI and, after consultations and modifications, were accepted by the local teams. In addition, the PI is available to consult with the teams, respond to local issues and questions, collaborate with the MHCC, and help to ensure conformity of evaluation measures across sites through participation in an ongoing community of practice involving all the research coordinators. Besides group consultations during research community of practice meetings, one-on-one consultations are available by email, telephone, and video conferencing whenever requested by researchers from local sites.

Since it is important to also obtain qualitative data from interviews and focus groups involving the people in charge of the implementation and the evaluation, the role of doing this was delegated to the PI, with MHCC providing some additional necessary resources made available to local researchers (software licenses and transcription services). This is because it could be difficult for employees working for a research or implementation coordinator to conduct a completely nonbiased interview with one of their supervisors or employers. In addition to locally obtained data, the PI team will obtain some pertinent centralized national data to support and share with local research teams. 

The local research teams, hired by the local organization responsible for the implementation, are responsible for data collection and analyses in their communities, and they provide local reports and summaries that are requested or needed by those involved in the local implementation. They have a mandate to collaborate with the PI to determine the research methods, as summarized in this document, and participate in a community of practice with the PI and other RoH community researcher coordinators. A data sharing agreement has been developed to specify ethical and practical aspects of sharing and use of data by local researchers, the PI, and the MHCC. 

The PI also has the roles, mandated by the MHCC and agreed to by all the local research coordinators, of summarizing the data from each of the local research teams, pooling data when possible, conducting analyses on pooled data, drawing overall conclusions concerning the RoH model and specific activities that exist at multiple sites, discussing site-specific aspects of the implementation and effects, and presenting potential implications of the evaluation results for scaling-up and implementation in different sites and contexts.

## Methods

### Ethics Approval

This evaluation protocol has been approved by the Ethics Committee for Research with Human Subjects (“Comité institutionnel d’éthique de la recherche avec des êtres humaines”) of the Université du Québec à Montréal (dossier number 2758).

### Participating Communities

The initial sites of the implementation of RoH were Newfoundland and Labrador, New Brunswick, Saskatchewan (3 sites), Alberta, Ontario, and Nunavut. The first province to become part of the RoH project was Newfoundland and Labrador, which partnered with the Mental Health Commission in January 2018. This collaboration was developed by Eastern Health and a community coalition on the Burin Peninsula. The province of Newfoundland and Labrador was joined by New Brunswick, which launched a community suicide prevention project in Northwestern New Brunswick in the County of Madawaska-Victoria, which includes Kedgwick, Edmundston, and Grand Falls. Three additional provinces (Sakatchewan, Alberta, and Ontaria) also joined RoH. In Saskatchewan, with partnerships between local community coalitions and the Saskatchewan Health Department, the project was implemented in the Northern communities of La Ronge and Meadow Lake. The community of Buffalo Narrows, Saskatchewan joined in 2020. In Alberta, RoH was implemented in Edmonton as part of the Living Hope community suicide prevention of that city, in collaboration with local organizations and a partnership with the Canadian Mental Health Association. The Waterloo-Wellington, Ontario community signed on to RoH in August 2019, with the Waterloo Region Suicide Prevention Council leading this initiative and partnering with Here4Hope, the Wellington Suicide Prevention Initiative involving the Canadian Mental Health Association Waterloo-Wellington and Wellington County. The city of Iqaluit in the Canadian Teritory of Nunavut joined RoH in 2021 as part of the Department of Health of the Government of Nunavut’s Inuusivut Anninaqtua Action Plan for suicide prevention. At the time this evaluation plan was submitted, Iqaluit had not yet completed its planning phase and had not yet started implementation and evaluation activities, partly due to the COVID-19 pandemic.

### Theory of Change and Evaluation Model 

**Figure 1:**
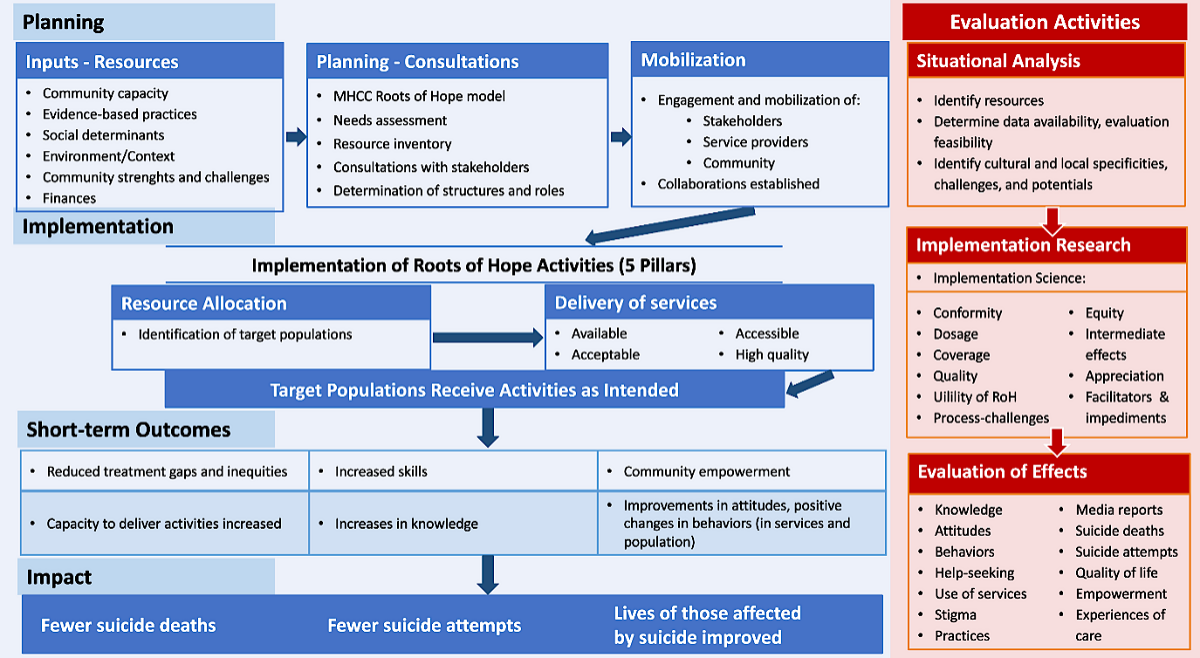
Roots of Hope (RoH) theory of change and evaluation model. MHCC: Mental Health Commission of Canada.

A theory of change is intended to generate “a description of a sequence of events that is expected to lead to a particular desired outcome” [6]. Figure 1 presents a diagram with the theory of change and evaluation model for the implementation and evaluation of the RoH project. A theory of change, as represented in a diagram and narrative, aims to provide a guiding framework for the project team and stakeholders. The theory of change presented in Figure 1 is the result of a dynamic iterative process, based upon the study of documents provided by the MHCC and the PI, and discussions with the persons conducting the RoH program in each of the provinces and the provincial research coordinators, as well as the consideration of provincial evaluation proposals and local resources. The model in Figure 1 follows a logical sequential order, from the top to the bottom, with parallel evaluation activities being conducted at each step.

The evaluation model begins with an assessment of the planning, which occurred before implementation of the RoH activities and continues to be adjusted throughout the implementation of the activities. Changing realities oblige everyone involved in planning, implementation, and evaluation to constantly adapt what they are doing, based upon how they conceive of changing needs, priorities, and responses to their actions in the flux of change in complex real-life situations. For this reason, a theory of change has been described as both a process and a product that continually evolves over time [6]. 

The planning is based upon the community resources, strengths, and challenges, and the sociocultural community context. Furthermore, what constitutes evidence-based practices in suicide prevention is an important resource input to help guide planning of activities. In an ideal situation, before planning starts, one should begin with a needs assessment to understand the magnitude, nature, and characteristics of the target population, their needs, the community needs, and available resources. Although each of the RoH communities discussed and considered needs and resources in the development of their specific RoH activities before the evaluation team was put in place, this was not always complete, and certain aspects of needs were assessed differently at each site. Therefore, the evaluation of RoH includes a standardized systematic situational analysis in each of the communities in order to better understand the local contexts in which the RoH activities were developed. This situational analysis, conducted at each of the sites using available and reliable data sources, identifies the nature of the psychosocial context in the communities, the community resources and capacities, and the challenges within the community at the general level and for specific cultural and local subgroups, including particular populations at increased risk of suicidal behaviors.

Multimedia Appendix 2 lists the information gathered as part of the situational analysis. Besides describing the communities for use in local planning, the PI team summarized this information across communities in order to provide a portrait of similarities and differences. This comparative portrait can help in the interpretation of evaluation results by identifying common issues, strengths, and resources, as well as the specificities associated with each of the sites.

Planning in each site also involved consultations with stakeholders, structuring of roles, and concrete assessments of needs and resources. All this was considered in the context of the RoH model that was developed by the MHCC, which constitutes the basic framework according to which each community is expected to develop their local plan of activities.

The planning process in each community involves mobilization of stakeholders, service providers, and members of the community to become involved in both the planning and implementation activities of RoH. In each of the communities, collaborations need to be established. This was generally concretized in the form of local coordinating and planning committees, as well as community consultations to not only provide information about what to do and how to do it, but also develop active engagement by individuals, organizations, and community agencies for realizing the RoH project. 

The planning phase, whose documentation is part of the RoH implementation evaluation, is translated in each site into specific implementation activities organized into the 5 “pillars” of RoH (Multimedia Appendix 2) as specified by the RoH model developed by the MHCC. For each activity, the nature and extent of resources allocated for each of the target populations, as well as the nature of the activities, need to be determined in relation to the community resources. Then, the delivery of services can begin. Characteristics of these services should be understood as part of the evaluation process in terms of their availability, accessibility, acceptability, and quality. In an ideal world, this results in the target populations receiving the activities as planned and intended. However, in reality, not everything can be implemented as planned, and multiple adjustments and adaptations need to be made throughout the implementation phase.

During this implementation phase, we assess important characteristics of the implementation of RoH activities in each of the pillars as follows: *conformity* in providing activities as planned; *dosage* (extent to which enough of the service or activity was effectively delivered); *coverage* (proportion of the general population and targeted populations reached); *quality* (degree to which activities and services were of high quality); *equity* (extent to which coverage was biased due to some systemic characteristics such as location and accessibility issues); *appreciation* (how services and activities were perceived and received by stakeholders and end users); and *conformity* to best practices in suicide prevention and to the RoH model (evidence based). 

In addition, we focus on identifying facilitators that help with “successful” implementation and help the persons involved in the implementation address challenges and difficulties throughout the implementation process. 

Although much of the RoH evaluation focuses on implementation, to the extent possible, the evaluation model includes assessments of short-term outcomes, and mid-term impacts and effects. As shown in Figure 1, the RoH evaluation focuses on the following 7 primary areas of short-term outcomes: (1) reductions in treatment gaps and inequities, (2) increases in skills, (3) community empowerment, (4) increases in capacity to deliver activities, (5) increases in knowledge, (6) improvements in attitudes, and (7) positive changes in behaviors in providing services and the behaviors of members of the population. 

The evaluation of effects is both specific to different RoH activities and general, in the sense that it also concerns the synergistic effects of the combination of all RoH activities in each community. Figure 1 lists the 12 main areas of the evaluation of effects. Most of these areas can be assessed in the short term regarding specific activities and more generally in the mid-term regarding the impact on the community. For example, evaluation of knowledge after participating in a training session can be assessed among participants in the session. However, a combination of multiple training and educational activities within the community could result in less stigma and negative attitudes about help-seeking. This, in turn, could facilitate increased use of mental health and suicide prevention services, and could have an impact on the prevention of suicidal behaviors. 

Because of the low incidence of suicide deaths and the fact that suicide rates can have large fluctuations in relatively small communities, one cannot expect to observe significant decreases in suicide deaths over a short period (implementation period of approximately 2 years) and to reliably associate those changes with RoH. However, it is possible that pooling data across all RoH communities could indicate trends in suicide rates a few years after the implementation of RoH. Since there are far more suicide attempts than suicide deaths, if data are available, one may be able to document changes in suicide attempts in a shorter period of time. To the extent possible, RoH will examine existing data on attempted suicide. However, systematic suicide attempt data are not compiled in an exhaustive or accurate manner across Canada. Hospital records and visits to private physicians are rarely complete. Furthermore, a complete portrait of the incidence of suicide attempts in a community must include information from all individuals and institutions who have contacts with persons who have attempted suicide, which is often difficult and impractical. Again, we will analyze what data are available to determine if there is sufficient information to indicate changing trends in the rates of attempted suicide. 

One can also assess the extent to which the lives of those affected by suicide may be improved as a result of RoH activities. Persons affected by suicide include those who are feeling suicidal and are in need of help; those at high risk of suicide who may or may not proceed with a suicide attempt; those who have attempted suicide and are at greater risk of attempting again; and the family, friends, and co-workers of persons who are thinking of suicide, who have attempted suicide, or who have died by suicide.

### Methodologies and Measures

The methodologies and measures used to answer the major evaluation questions are listed in Multimedia Appendices 3-11. It is to be noted that all common measures proposed and listed are the result of a lengthy process of consultation and collaboration between the PI team and local researchers. They are to be understood as constituting a minimum set of information and data to be obtained in order to inform the implementation process of RoH as a whole. However, as explained below, this has proven to be particularly difficult in light of changing local contexts and resource limitations. Therefore, individual teams are encouraged throughout the process to make decisions on the most pertinent measures to retain in accordance with their local focus and the realities of local capacity. Nonetheless, all decisions concerning local measurements should be systematically documented and explained. 

Although it seems desirable to determine which methodologies and instruments to use in advance and then simply proceed to gather the information, an implementation science approach obliges researchers to be constantly aware of the practicalities involved. They should consider the extent to which the instruments and methodologies they planned to use actually provide accurate and useful information that they require within the constraints of local contexts (research acceptability, availability of reliable data, resources, and capacity). Therefore, successful evaluations are iterative processes involving continued mindfulness of how well the evaluation is going at every step, and an openness to making changes and improvements throughout the process. The preceding statement proved particularly pertinent to the evaluation of RoH in the context of the COVID-19 pandemic. The COVID-19 pandemic occurred just as implementation of RoH was beginning in most communities. Not only were many activities and evaluation components put on hold during the COVID-19 pandemic, but also creative adaptations and changes were often made to continue some activities in other formats and adjust priorities in the identification of vulnerable populations. During this period, information about the changes associated with the COVID-19 pandemic and their impacts was systematically gathered as part of this evaluation. Multimedia Appendix 3 shows the information gathered and methodologies to determine what factors could affect the planning components of the RoH model in different contexts as well as overall. Data on planning are obtained from documents and the situational analysis (Multimedia Appendix 2 and the discussion in the following section). However, the evaluation of the planning components is complemented by qualitative data from interviews with each of the community coordinators and coordination groups, stakeholder focus groups, interviews with key RoH personnel, persons with lived experiences, and other available local data.

One of the key issues involves the extent to which the implementation of activities and services is both adequate and equitable. This involves not only an assessment of the extent to which each component is implemented, but also an understanding of key target populations in each community and the extent to which RoH activities provide interventions to various subgroups in a manner that meets their needs. Some of the measures concern the nature of the implementation process (conformity, dosage, quality, and coverage). However, a true understanding of adequate and equitable implementation involves including an assessment of the adequacy of the activities in meeting the needs of different target populations, and their real and perceived impacts (Multimedia Appendix 4). 

Each of the RoH communities provide multiple training activities to care providers. Care providers include not only professionals, such as general practice physicians and workers at crisis centers, but also members of the community, such as those receiving peer-support training in a workplace. Multimedia Appendix 5 lists the data sources for evaluating training activities. These include descriptions of the nature and extent of training activities, participants, quality assessments of sessions and activities, and indications of the impact of specific training activities in increasing knowledge, improving attitudes, and increasing positive helpful behaviors.

The nature of awareness activities in this RoH awareness pillar varies greatly among communities, because of different target populations, community resources, and needs. Besides assessing activities to increase awareness and knowledge in the general population, several communities are also conducting general surveys to assess changes in stigma associated with suicide and mental health, attitudes, knowledge, and openness to seeking help (Multimedia Appendix 6).

The specialized support pillar of RoH includes a wide variety of specific services provided, expanded, or improved as part of RoH. The evaluation of these specialized support activities varies according to which activities are initiated in each community. For example, when decreasing wait times to obtain mental health services is a specific focus, the extent that wait times decrease needs to be assessed. When specific activities and programs are provided to help people bereaved by suicide, specific assessments need to be made to evaluate their impact on program participants. As seen in Multimedia Appendix 7, quantitative data using questionnaires and survey instruments are complemented by qualitative data from interviews and focus groups with key informants, clients, and service providers.

Reducing access to potential means for suicide is a RoH component whose activities depend upon which suicide methods are used or increasing in each community. Although it is possible to assess the roll out of these activities, and their nature and quality, most prior evaluations of promoting means safety have involved assessing decreases in deaths by suicide and suicide attempts. It is not realistic to expect that we would have sufficient data on suicide deaths to link those data to the targeted means safety activities in RoH communities. However, as indicated in Multimedia Appendix 8, we can assess the availability, acceptability, accessibility, and quality of the means safety activities and their coverage of target populations.

When we look at potential effects, the RoH evaluation model includes “classic” measures of suicidal behaviors (deaths, attempts, and possibly some information on the experiences of people with lived experience). However, as already discussed, the low incidence of suicide in general, lack of sufficient valid data on suicide attempts, relatively small population base in RoH communities, and limited time frame of the evaluation make it difficult to evaluation the impact of RoH on suicidal behaviors. Still, we will examine changes in the available data. However, we will also examine changes in other outcome measures that have been associated with decreases in suicidal behaviors in past research (Multimedia Appendix 9). These include improvements in services and changes in community perceptions and beliefs. Multimedia Appendix 10 provides several data sources for obtaining information on suicidal behaviors, as well as improvements that may be associated with fewer suicides and suicide attempts (community focus groups and surveys; administrative information and media reports; improvements in delivery capacity; identification of treatment gaps and inadequacies; and improvements in the skills, knowledge, and behaviors of service providers).

Because of the nature of community involvement throughout the process of planning, implementing, and evaluating RoH, we could expect increased community empowerment and quality of life, as well as improvements in perceptions of services and a diminution of barriers to accessing help. Decreasing stigma is one of the goals in each of the RoH communities that will be assessed. Increasing help-seeking behaviors, improving attitudes toward help-seeking, and increasing knowledge of services are the focuses of many of the RoH community interventions (Multimedia Appendix 10). 

### Data Analyses and Synthesis 

The local evaluation research coordinators at each site will be responsible for conducting analyses of their quantitative and qualitative data and producing synthesis reports for local use and reports to the MHCC (Multimedia Appendix 11). The PI will be available to consult and assist local coordinators with their analyses and interpretation of results. The PI team will also propose guidelines for common coding and analysis to ensure consistency across sites. All results of the measures indicated in this plan, as well as additional measures used as part of the RoH evaluation, will be shared with the PI, including the necessary information needed to summarize results across sites. The PI will conduct additional analyses combining information from sites, synthesize and interpret the overall results, and provide reports in collaboration with all contributing local researchers. After consultations, reports combining and comparing information for the sites will be included in reports by the PI. All data sharing will observe the norms and procedures outlined in the Data Sharing Agreement signed by the provincial partners, the PI and his university, and the MHCC. All publications will follow the Publication and Dissemination Guidelines for RoH developed by the MHCC in consultation with the PI, provincial partners, and research coordinators. 

Local research teams that could benefit from support and consultation in their analyses of qualitative data can call upon the PI and his team to help and support them with these analyses and their interpretation. The data gathered from the interviews and focus groups conducted by the PI’s team at local sites will be analyzed by the PI team, and the results will be sent to the provincial research coordinators for use in their reports. The results combined across sites may be used in overall RoH reports and presentations produced by the PI and his team, in accordance with the Data Sharing Agreement and the Publication and Dissemination Guidelines, approved by the MHCC, the PI, and all local research coordinators.

## Results

The evaluation research plan for RoH is a major initiative involving diverse sites across Canada, each implementing a variety of multifaceted suicide prevention–related activities to meet the local needs of various target populations. The RoH model, developed by the MHCC, includes “state-of-the-art” best practices that have been validated in prior research and are recommended by the WHO to be incorporated in national suicide prevention strategies. This model provides a general orientation to focus on 5 “pillars” of action, leaving it up to each site to engage in consultation, planning, and evaluation processes that take into account local problems, resources, and cultures, and the specific needs of priority target populations that are identified. In this context, one would expect that evaluations of the implementation and effects would need to be tailored to the local needs and resources, and the nature of the specific activities selected to be implemented as part of RoH at each site. Therefore, the evaluation model must carefully balance the need for standardization and the advantages of using common approaches and methodologies, against the necessity to tailor the evaluation activities at each site to the unique characteristics of the RoH activities that have been chosen. In this context, the extent to which the evaluation results across the diverse communities will be able to identify valid general conclusions that are applicable to current and future RoH sites will not be determined until the evaluation is completed.

The evaluation model presented in this document demonstrates the fruits of over 2 years of interactions between the PI team and local research and implementation coordinators, with input from the MHCC. Hopefully, this plan attains a balance between standardization and flexibility that will provide the necessary information to meet local needs at each site and also provide a comprehensive overall assessment of the RoH model across sites. However, differences in the nature and extent of funding at each site and differences in the availability of data in existing community organizations and records may compromise the ability of the evaluation to obtain enough of the essential information necessary to attain the evaluation goals.

We realize that real-world constraints and changes during the course of the RoH project will continually pose challenges to all those involved in the evaluation process. The COVID-19 pandemic is a powerful example of how radical changes in the planned roll out of the programs and evaluation activities had to be made, including refocusing and developing new activities to respond to suicide and mental health challenges associated with the pandemic, and changing data collection methods for the evaluation. These changes primarily concerned the need to cancel many in-person activities and replace them with online programs, services, and assessments. The advent of COVID-19 during the implementation phase resulted in the need to innovate and adapt, and caused delays in the implementation and evaluation processes. However, this also provided an opportunity to understand the process of adapting to the challenges of having COVID-19 present in communities, where new methods were developed to deliver services and to obtain data for the evaluation.

The quantitative and qualitative data from all sites will be summarized by the PI in March 2023 to draw conclusions to help the MHCC in its improvements to the RoH model, and to inform communities about how to better implement the RoH approach in an efficacious and equitable manner. Since the COVID-19 pandemic occurred at the beginning of program implementation, its impact on the implementation and evaluation activities and its influence on the RoH project will be documented. Overall, we aim to clarify the validity of RoH in contributing to the prevention of suicides and suicidal behaviors in a variety of contexts. The final evaluation report will be available in September 2023.

## Discussion

Our aspiration for the evaluation is to be able to better understand the validity, feasibility, and evaluability of the RoH model for community suicide prevention. We also aspire to identify the processes in planning, implementation, and evaluation that facilitate its usefulness for communities and their members. We hope to clarify its validity in contributing to the prevention of suicides and suicidal behaviors in a variety of contexts. We also hope that it may help others who are interested in developing suicide prevention strategies and activities, including those who are considering adopting the RoH model. In addition, the evaluation results may provide useful information to the MHCC on how RoH could be modified and improved to be more effective in the future. 

Besides reaching general conclusions about the practicality and usefulness of the RoH model for communities, we hope to be able to identify factors that facilitate its implementation, factors that inhibit its implementation, and different adaptations and innovations that can be used to deal with challenges occurring during its implementation. We aim to identify the extent to which the implementation is successful and equitable, and explain why problems may have arisen, what facilitates success, and how the Mental Health Commission may be able to modify the RoH model to facilitate the implementation, equity, and positive effects of the program.

Although the evaluation is not complete, challenges in coordinating locally funded evaluation programs in multiple sites are evident. A better future approach could be to obtain sufficient central funding for the entire implementation and evaluation. This would better ensure that all sites have the necessary resources to complete all the essential evaluation activities and would minimize discrepancies between sites in which activities are implemented completely as planned. In a provincial context, such as Canada, where health and mental health services are planned, funded, and delivered by provinces, it may not be feasible to ensure uniform funding for the implementation of community programs for sites in different provinces. However, since there are national research funding organizations, there is hope that multisite evaluations of the implementation and short-term effects could be funded.

## Appendix

Multimedia Appendix 1 Mental Health Commission of Canada aspirational questions.

Multimedia Appendix 2 Situational analysis.

Multimedia Appendix 3 Assessing planning: factors affecting planning components, methodologies, and sources of data.

Multimedia Appendix 4 Assessing implementation: adequacy of services and activities to target populations and equity issues, methodologies, and sources of data.

Multimedia Appendix 5 Training pillar: implementation common metrics, methodologies, and sources of data.

Multimedia Appendix 6 Awareness pillar: implementation common metrics, methodologies, and sources of data.

Multimedia Appendix 7 Specialized support pillar: implementation common metrics, methodologies, and sources of data.

Multimedia Appendix 8 Means restriction pillar: implementation common metrics, methodologies, and sources of data.

Multimedia Appendix 9 Short-term outcomes, methodologies, and sources of data.

Multimedia Appendix 10 Impact on suicidal behaviors, quality of life, community empowerment, experience of care, and effects on practices.

Multimedia Appendix 11 Summary of common measures and assessments for quantitative and qualitative data.

## Abbreviations

MHCC: Mental Health Commission of Canada
PI: principal investigator
RoH: Roots of Hope
WHO: World Health Organization

## Data Availability

Raw data gathered by the principal investigator that are not reported in the final report of the evaluation research may be obtained (anonymized) by contacting the principal investigator once the report and articles reporting the findings have been published. Data gathered locally at each of the sites, which will be synthetized or interpreted by the principal investigator in the final report, will be made available according to the policies of each of the local research coordinators who obtained the data.
