# Supplementary material for: Evaluation of a Community Suicide Prevention Project (Roots of Hope): Protocol for an Implementation Science Study
Source: JMIR Res Protoc. 2023 Jun 14;12:e39978. doi: 10.2196/39978 (PMC10337351; doi:10.2196/39978)
Supplement: Multimedia Appendix 1 [file resprot_v12i1e39978_app1.docx]

**Multimedia Appendix 1.** Mental Health Commission of Canada aspirational questions.

| Roots of Hope Model  1. Is the Roots of Hope model a comprehensive model to reduce the impacts of suicide within communities across Canada? E.g.  a. Does it have appropriate pillars?  b. Does it require additional pillars?  2. What elements are perceived by communities as being essential to implement the model?    Formative evaluation  1. How did communities implement the Roots of Hope model? (i.e. steps taken)  2. To what degree did each of the communities follow the Roots of Hope model?  3. What adaptations were made to tailor the model to the local context?  4. What were their facilitators to success?  5. What were the barriers to implementation?  6. What were the overall lessons learned that would be useful to share with other communities implementing the model?  7. To what extent was the MHCC’s support to the communities useful in supporting their implementation journey?  a. What support is needed by communities to implement the Root of Hope model?  8. What is the program logic model for the Root of Hope approach?  a. What are some of the short, medium, and long term expected outcomes from the perspective of the process of implementation?  i. To what extent were targeted outcomes of the Roots of Hope model perceived to have been achieved?  9. How did the COVID pandemic impact the implementation journey of the communities?  10. To what degree the Roots of Hope model was implemented in an equitable manner?    Outcome evaluation  1. What is the perceived impact of the successful implementation of the Roots of Hope model on:  a. Access to services  b. Improved client experience of care  c. Suicide rates and behaviours  2. To what extent is the impact of the Roots of Hope project perceived to yield different impacts based on gender, age, ethnic-racial status, geography, socio-economic status?  3. How is the COVID perceived as impacting the expected outcomes of the project? |
| --- |
| Other questions     1. What are the expected implications of the Roots of Hope project for future National suicide prevention policies? |
