# Supplementary material for: Evaluation of a Community Suicide Prevention Project (Roots of Hope): Protocol for an Implementation Science Study
Source: JMIR Res Protoc. 2023 Jun 14;12:e39978. doi: 10.2196/39978 (PMC10337351; doi:10.2196/39978)
Supplement: Multimedia Appendix 2 [file resprot_v12i1e39978_app2.docx]

## **Multimedia Appendix 2.** Situational analysis.

## Situational Analysis Overview

A situational analysis is the first step in assessing needs of your community. It is a systematic process of gathering, analyzing, synthesizing and communicating available data about relevant facts, such as the epidemiology, demography and health status of the population. It should also include relevant information about the current situation as compared to expectations. Thus, according to World Health Organization (2012), it might include:

- social determinants of health and health needs, such as income and education
- expectations
- health system performance and of performance gaps in responding to needs and expectations
- capacity of the health sector and community resources to respond to current and to anticipate future challenges
- health system and community resources (human, physical, financial, informational) and of resource gaps in responding to needs and expectations
- stakeholders’ positions (including, where appropriate, positions of external partners).

This systematic process of gathering and analyzing data about the current situations of concern as well as existing resources can serve as the basis to specify the scope of the problem and its distribution. It can help determine priorities in planning by identifying areas of possible impact and help to identify variables to be examined as part of an evaluation of the implementation of programs and their effects.

### Sources of data

Reliable sources may include:

- Statistics Canada
- Royal Canadian Mounted Police (RCMP) data
- Canadian Coroner and Medical Examiner ([CCMED common data set](http://www23.statcan.gc.ca/imdb/p2SV.pl?Function=getSurvey&SDDS=5125))
  - [Alberta’s Office of the Chief Medical Examiner](https://www.alberta.ca/office-of-chief-medical-examiner.aspx)
  - [Saskatchewan Coroners Services](https://www.saskatchewan.ca/government/government-structure/boards-commissions-and-agencies/saskatchewan-coroners-service)
  - [Manitoba’s Office of the Chief Medical Examiner](https://www.gov.mb.ca/justice/crown/cme.html)
  - [Ontario’s Office of the Chief Coroner](http://www.mcscs.jus.gov.on.ca/english/DeathInvestigations/office_coroner/coroner.html)
  - [Services des coroners du Nouveau-Brunswick](https://www2.gnb.ca/content/gnb/fr/services/services_renderer.14198.Services_des_coroners.html)
  - [Newfoundland-Labrador’s Office of the Chief Medical Examiner](https://www.justice.gov.nl.ca/just/department/branches/division/division_ocme.html)
- [Canadian Institute for Health Information](https://www.cihi.ca/en) (CIHI) (provincial hospitalization data by cause)
- Agency records
- Municipal and provincial surveys and censuses
- Municipal and provincial reports
- Focus groups
- Key informants

It is important to clearly indicate sources of data.

It is important to complete all sections listed below to the extent possible. Since data reliability and accuracy vary according to the source of information, please always indicate the source.

## Contextual factors

Population needs and effective solutions depend on contextual factors. These are factors that influence the service environment as well as the receptivity of the general population and stakeholders to suicide prevention initiatives. Although not always quantifiable, a careful description of those factors will help to identify areas of weaknesses and potential facilitators in your community.

|  | **Description**  **Include quantitative data when available** | **Strengths** | **Weaknesses or challenges** |
| --- | --- | --- | --- |
| **Physical factors** | | | |
| Rural or urban location | insert  Indicate source | insert  Indicate source | insert  Indicate source |
| Population and population density | insert  Indicate source | insert  Indicate source | insert  Indicate source |
| Barriers and facilitators to access to services (e.g., great distances) | insert  Indicate source | insert  Indicate source | insert  Indicate source |
| *Other* | insert  Indicate source | insert  Indicate source | insert  Indicate source |

| **Transportation and mobility** | **Description**  **Include quantitative data when available** | **Strengths** | **Weaknesses or challenges** |
| --- | --- | --- | --- |
| Average distances to services | insert  Indicate source | insert  Indicate source | insert  Indicate source |
| Public transit ( accessible transportation, coverage, cost) | insert  Indicate source | insert  Indicate source | insert  Indicate source |
| Traffic concerns | insert  Indicate source | insert  Indicate source | insert  Indicate source |
| Weather issues | insert  Indicate source | insert  Indicate source | insert  Indicate source |
| Snow removal, road maintenance | insert  Indicate source | insert  Indicate source | insert  Indicate source |
| Issues relating to climate change or natural disasters | insert  Indicate source | insert  Indicate source | insert  Indicate source |
| *Other* | insert  Indicate source | insert  Indicate source | insert  Indicate source |

| **Infrastructure and quality of life** | **Description**  **Include quantitative data when available** | **Strengths** | **Weaknesses or challenges** |
| --- | --- | --- | --- |
| Health care facilities (list, describe briefly and indicate population served, include quantitative data from reports) | insert  Indicate source | insert  Indicate source | insert  Indicate source |
| Emergency services (fire, police, ambulance, include quantitative data from reports) | insert  Indicate source | insert  Indicate source | insert  Indicate source |
| Educational facilities (list, indicate population served, describe workforce and provide quantitative data from reports) | insert  Indicate source | insert  Indicate source | insert  Indicate source |
| Community, artistic and cultural buildings and facilities, including parks (describe, and describe use levels and levels of community participation) | insert  Indicate source | insert  Indicate source | insert  Indicate source |
| Access to housing and affordability (narrative description, including data if available) | insert  Indicate source | insert  Indicate source | insert  Indicate source |
| Housing policies (special needs housing, rent control, housing subsidies, etc.) | insert  Indicate source | insert  Indicate source | insert  Indicate source |
| Types of employment  Employment conditions (e.g. unemployment, precarious employment, informal employment, etc.)  Working conditions (e.g. injuries, hazards, psychosocial stressors) | insert  Indicate source | insert  Indicate source | insert  Indicate source |
| Food security and safety (data from Health Canada when available for your area)  Access to clean water | insert  Indicate source | insert  Indicate source | insert  Indicate source |
| Access to information and communication technology (describe ICT networks, including internet connection: universality, equity, affordability) | insert  Indicate source | insert  Indicate source | insert  Indicate source |
| *Other* | insert  Indicate source | insert  Indicate source | insert  Indicate source |

| **Health and mental health system** | **Description**  **Include quantitative data when available** | **Strengths** | **Weaknesses or challenges** |
| --- | --- | --- | --- |
| Leadership and governance (narrative descriptions, organigram if available) | insert  Indicate source | insert  Indicate source | insert  Indicate source |
| Information systems to access data on patients (describe, who has access, % of population covered) | insert  Indicate source | insert  Indicate source | insert  Indicate source |
| Health and Mental Health Workforce (recruitment, education, distribution, performance, retention, problems, strengths and challenges, quantitative data from reports when available) | insert  Indicate source | insert  Indicate source | insert  Indicate source |
| Service delivery : access and quality (narrative description, with data from reports if available, e.g. wait times) | insert  Indicate source | insert  Indicate source | insert  Indicate source |
| *Other* | insert  Indicate source | insert  Indicate source | insert  Indicate source |

| **Cultural environment** | **Description**  **Include quantitative data when available** | **Strengths** | **Weaknesses or challenges** |
| --- | --- | --- | --- |
| Local beliefs about causes of mental health problems and suicide (describe any particularities, including stigma and how this affects openness versus reluctance to discuss) | insert  Indicate source | insert  Indicate source | insert  Indicate source |
| Local beliefs about appropriate treatments (note any local beliefs, customs and practices) | insert  Indicate source | insert  Indicate source | insert  Indicate source |
| Stigma of mental illness and suicide | insert  Indicate source | insert  Indicate source | insert  Indicate source |
| Family characteristics (e.g., couples with or without children, children living with two parents or one parent, stepfamilies)  Values pertaining to family structure (e.g. cultural values that influence family’s structure, arrangement, function and roles of families’ members) | insert  Indicate source | insert  Indicate source | insert  Indicate source |
| Religions and spiritual beliefs | insert  Indicate source | insert  Indicate source | insert  Indicate source |
| Principal religions, extent of practice and participation | insert  Indicate source | insert  Indicate source | insert  Indicate source |
| Traditions (social customs, beliefs transmitted from generation to generation) | insert  Indicate source | insert  Indicate source | insert  Indicate source |

| Cultural diversity and presence of cultural groups, ethnic/visible minorities.  Specify. | insert  Indicate source | insert  Indicate source | insert  Indicate source |
| --- | --- | --- | --- |
| Neighbourhood cohesion | insert  Indicate source | insert  Indicate source | insert  Indicate source |
| *Other* | insert  Indicate source | insert  Indicate source | insert  Indicate source |

## Suicide indicators

These data are specific to deaths by suicide and suicide attempts. The objective is to identify measurable, verifiable and meaningful data capable of assessing trends over time. Combined with socio-economic indicators, they will provide key breakdowns in order to describe target populations in ways that can better orient the program implementation.

Whenever possible, retrieve the information **for the past ten years** according to age group and sex. It is very important to indicate clearly the source of the data. This will be useful to assess the methods of collecting data that had been used as well as the extent to which some data may be compared with others.

For example, data on suicide attempts can be obtained from hospital records, but some data on hospitalizations are also available from the [Canadian Institute for Health Information](https://www.cihi.ca/en) (CIHI).

For more information on the Coroner/Medical Examiner system, visit the [CCMED database](http://www23.statcan.gc.ca/imdb/p2SV.pl?Function=getSurvey&SDDS=5125).

**Very important: indicate sources of data, date of data and geographical area here:** insert

| Deaths by suicide | | | | | | | | | | | | | | | | | | | | |
| --- | --- | --- | --- | --- | --- | --- | --- | --- | --- | --- | --- | --- | --- | --- | --- | --- | --- | --- | --- | --- |
| **Year of data**  **10 most recent years with available data** | insert | | insert | | insert | | insert | | insert | | insert | | insert | | insert | | insert | | insert | |
| **Sex** | **M** | **F** | **M** | **F** | **M** | **F** | **M** | **F** | **M** | **F** | **M** | **F** | **M** | **F** | **M** | **F** | **M** | **F** | **M** | **F** |
| Rates (deaths per 100.000 people) | insert | insert | insert | insert | insert | insert | insert | insert | insert | insert | insert | insert | insert | insert | insert | insert | insert | insert | insert | insert |
| Numbers | insert | insert | insert | insert | insert | insert | insert | insert | insert | insert | insert | insert | insert | insert | insert | insert | insert | insert | insert | insert |
| *Sources of data* | insert | | insert | | insert | | insert | | insert | | insert | | insert | | insert | | insert | | insert | |
| **Method used as recorded by coroner or medical examiner (numbers)** | | | | | | | | | | | | | | | | | | | | |
| Drug poisoning | insert | insert | insert | insert | insert | insert | insert | insert | insert | insert | insert | insert | insert | insert | insert | insert | insert | insert | insert | insert |
| Gas poisoning | insert | insert | insert | insert | insert | insert | insert | insert | insert | insert | insert | insert | insert | insert | insert | insert | insert | insert | insert | insert |
| Other chemical poisoning | insert | insert | insert | insert | insert | insert | insert | insert | insert | insert | insert | insert | insert | insert | insert | insert | insert | insert | insert | insert |
| Hanging,  asphyxiation | insert | insert | insert | insert | insert | insert | insert | insert | insert | insert | insert | insert | insert | insert | insert | insert | insert | insert | insert | insert |
| Drowning | insert | insert | insert | insert | insert | insert | insert | insert | insert | insert | insert | insert | insert | insert | insert | insert | insert | insert | insert | insert |
| Firearm | insert | insert | insert | insert | insert | insert | insert | insert | insert | insert | insert | insert | insert | insert | insert | insert | insert | insert | insert | insert |
| Stabbing | insert | insert | insert | insert | insert | insert | insert | insert | insert | insert | insert | insert | insert | insert | insert | insert | insert | insert | insert | insert |
| Jumping | insert | insert | insert | insert | insert | insert | insert | insert | insert | insert | insert | insert | insert | insert | insert | insert | insert | insert | insert | insert |
| Others | insert | insert | insert | insert | insert | insert | insert | insert | insert | insert | insert | insert | insert | insert | insert | insert | insert | insert | insert | insert |
| *Sources of data* | insert | | insert | | insert | | insert | | insert | | insert | | insert | | insert | | insert | | insert | |
| **Age Category** | | | | | | | | | | | | | | | | | | | | |
| Below 15 years old | insert | insert | insert | insert | insert | insert | insert | insert | insert | insert | insert | insert | insert | insert | insert | insert | insert | insert | insert | insert |
| 15 – 39 years | insert | insert | insert | insert | insert | insert | insert | insert | insert | insert | insert | insert | insert | insert | insert | insert | insert | insert | insert | insert |
| 40 – 59 years | insert | insert | insert | insert | insert | insert | insert | insert | insert | insert | insert | insert | insert | insert | insert | insert | insert | insert | insert | insert |
| 60 and older | insert | insert | insert | insert | insert | insert | insert | insert | insert | insert | insert | insert | insert | insert | insert | insert | insert | insert | insert | insert |
| *Sources of data* | insert | | insert | | insert | | insert | | insert | | insert | | insert | | insert | | insert | | insert | |
| **Characteristics of the victim or precipitating events** | | | | | | | | | | | | | | | | | | | | |
| Previous suicide behavior | insert | insert | insert | insert | insert | insert | insert | insert | insert | insert | insert | insert | insert | insert | insert | insert | insert | insert | insert | insert |
| Unemployed or financial difficulties | insert | insert | insert | insert | insert | insert | insert | insert | insert | insert | insert | insert | insert | insert | insert | insert | insert | insert | insert | insert |
| Divorced, separated, widowed | insert | insert | insert | insert | insert | insert | insert | insert | insert | insert | insert | insert | insert | insert | insert | insert | insert | insert | insert | insert |
| Chronic mental illness | insert | insert | insert | insert | insert | insert | insert | insert | insert | insert | insert | insert | insert | insert | insert | insert | insert | insert | insert | insert |
| Debilitating physical illness | insert | insert | insert | insert | insert | insert | insert | insert | insert | insert | insert | insert | insert | insert | insert | insert | insert | insert | insert | insert |
| Drugs or alcohol involved | insert | insert | insert | insert | insert | insert | insert | insert | insert | insert | insert | insert | insert | insert | insert | insert | insert | insert | insert | insert |
| Social isolation | insert | insert | insert | insert | insert | insert | insert | insert | insert | insert | insert | insert | insert | insert | insert | insert | insert | insert | insert | insert |
| Violence, trauma or abuse | insert | insert | insert | insert | insert | insert | insert | insert | insert | insert | insert | insert | insert | insert | insert | insert | insert | insert | insert | insert |
| Social media involved | insert | insert | insert | insert | insert | insert | insert | insert | insert | insert | insert | insert | insert | insert | insert | insert | insert | insert | insert | insert |
| Veteran | insert | insert | insert | insert | insert | insert | insert | insert | insert | insert | insert | insert | insert | insert | insert | insert | insert | insert | insert | insert |
| *Others* | insert | insert | insert | insert | insert | insert | insert | insert | insert | insert | insert | insert | insert | insert | insert | insert | insert | insert | insert | insert |
| *Sources of data* | insert | | insert | | insert | | insert | | insert | | insert | | insert | | insert | | insert | | insert | |

| Attempted suicides | | | | | | | | | | | | | | | | | | | | |
| --- | --- | --- | --- | --- | --- | --- | --- | --- | --- | --- | --- | --- | --- | --- | --- | --- | --- | --- | --- | --- |
| **Year of data**  **10 most recent years with available data** | insert | | insert | | insert | | insert | | insert | | insert | | insert | | insert | | insert | | insert | |
| **Sex** | **M** | **F** | **M** | **F** | **M** | **F** | **M** | **F** | **M** | **F** | **M** | **F** | **M** | **F** | **M** | **F** | **M** | **F** | **M** | **F** |
| **Hospitalizations related to attempted suicide and self-inflicted injury (by numbers and/or rates – preferably both)** | | | | | | | | | | | | | | | | | | | | |
| Total | insert | insert | insert | insert | insert | insert | insert | insert | insert | insert | insert | insert | insert | insert | insert | insert | insert | insert | insert | insert |
| Below 15 years old | insert | insert | insert | insert | insert | insert | insert | insert | insert | insert | insert | insert | insert | insert | insert | insert | insert | insert | insert | insert |
| 15 – 39 years old | insert | insert | insert | insert | insert | insert | insert | insert | insert | insert | insert | insert | insert | insert | insert | insert | insert | insert | insert | insert |
| 40 – 59 years old | insert | insert | insert | insert | insert | insert | insert | insert | insert | insert | insert | insert | insert | insert | insert | insert | insert | insert | insert | insert |
| 60 years and older | insert | insert | insert | insert | insert | insert | insert | insert | insert | insert | insert | insert | insert | insert | insert | insert | insert | insert | insert | insert |
| *Sources of data* | insert | | insert | | insert | | insert | | insert | | insert | | insert | | insert | | insert | | insert | |
| **Method used to attempt suicide or to self-harm** | | | | | | | | | | | | | | | | | | | | |
| Drug poisoning | insert | insert | insert | insert | insert | insert | insert | insert | insert | insert | insert | insert | insert | insert | insert | insert | insert | insert | insert | insert |
| Gas poisoning | insert | insert | insert | insert | insert | insert | insert | insert | insert | insert | insert | insert | insert | insert | insert | insert | insert | insert | insert | insert |
| Other chemical poisoning | insert | insert | insert | insert | insert | insert | insert | insert | insert | insert | insert | insert | insert | insert | insert | insert | insert | insert | insert | insert |
| Hanging,  asphyxiation | insert | insert | insert | insert | insert | insert | insert | insert | insert | insert | insert | insert | insert | insert | insert | insert | insert | insert | insert | insert |
| Drowning | insert | insert | insert | insert | insert | insert | insert | insert | insert | insert | insert | insert | insert | insert | insert | insert | insert | insert | insert | insert |
| Firearm | insert | insert | insert | insert | insert | insert | insert | insert | insert | insert | insert | insert | insert | insert | insert | insert | insert | insert | insert | insert |
| Stabbing | insert | insert | insert | insert | insert | insert | insert | insert | insert | insert | insert | insert | insert | insert | insert | insert | insert | insert | insert | insert |
| Jumping | insert | insert | insert | insert | insert | insert | insert | insert | insert | insert | insert | insert | insert | insert | insert | insert | insert | insert | insert | insert |
| Others | insert | insert | insert | insert | insert | insert | insert | insert | insert | insert | insert | insert | insert | insert | insert | insert | insert | insert | insert | insert |
| *Sources of data* | insert | | insert | | insert | | insert | | insert | | insert | | insert | | insert | | insert | | insert | |
| Follow-up after hospital discharge | | | | | | | | | | | | | | | | | | | | |
| With referrals | insert | insert | insert | insert | insert | insert | insert | insert | insert | insert | insert | insert | insert | insert | insert | insert | insert | insert | insert | insert |
| With physician follow-up | insert | insert | insert | insert | insert | insert | insert | insert | insert | insert | insert | insert | insert | insert | insert | insert | insert | insert | insert | insert |
| Without known follow-up care | insert | insert | insert | insert | insert | insert | insert | insert | insert | insert | insert | insert | insert | insert | insert | insert | insert | insert | insert | insert |
| *Sources of data* | insert | | insert | | insert | | insert | | insert | | insert | | insert | | insert | | insert | | insert | |

| Clustering of suicidal behaviour | | | | | | | | | | |
| --- | --- | --- | --- | --- | --- | --- | --- | --- | --- | --- |
| Hot-spots (describe) | insert  Indicate source | insert  Indicate source | insert  Indicate source | insert  Indicate source | insert  Indicate source | insert  Indicate source | insert  Indicate source | insert  Indicate source | insert  Indicate source | insert  Indicate source |
| Provide details on why you think this is a hot-spot | insert  Indicate source | | | | | | | | | |
| High risk time of the year | insert  Indicate source | insert  Indicate source | insert  Indicate source | insert  Indicate source | insert  Indicate source | insert  Indicate source | insert  Indicate source | insert  Indicate source | insert  Indicate source | insert  Indicate source |
| Provide details on why you think this particular time of year is high-risk | insert  Indicate source | | | | | | | | | |

| Places where suicides occur (if known. workplaces, schools, etc.) | insert  Indicate source | insert  Indicate source | insert  Indicate source | insert  Indicate source | insert  Indicate source | insert  Indicate source | insert  Indicate source | insert  Indicate source | insert  Indicate source | insert  Indicate source |
| --- | --- | --- | --- | --- | --- | --- | --- | --- | --- | --- |
| Presence of suicide clusters (multiple suicidal behaviours within an accelerated time frame) | insert  Indicate source | insert  Indicate source | insert  Indicate source | insert  Indicate source | insert  Indicate source | insert  Indicate source | insert  Indicate source | insert  Indicate source | insert  Indicate source | insert  Indicate source |

## Problems, challenges and expectations

| **Area of potential social issues** | **Understanding of the problem and its potential relation to suicide.**  **Provide quantitative data when available.** | **Who are confronted with the challenges? When? Where?**  **Provide quantitative data when available.** | **What are the current resources and potential facilitators? What are the protective factors?** | **What should be done?** |
| --- | --- | --- | --- | --- |
| **Economic inequality and poverty** | insert  Indicate source or key persons | insert  Indicate source or key persons | insert  Indicate source or key persons | insert  Indicate source or key persons |
| **Education and literacy** | insert  Indicate source or key persons | insert  Indicate source or key persons | insert  Indicate source or key persons | insert  Indicate source or key persons |
| **Health inequalities** | insert  Indicate source or key persons | insert  Indicate source or key persons | insert  Indicate source or key persons | insert  Indicate source or key persons |
| **Homelessness, poor housing** | insert  Indicate source or key persons | insert  Indicate source or key persons | insert  Indicate source or key persons | insert  Indicate source or key persons |
| **Drug abuse** | insert  Indicate source or key persons | insert  Indicate source or key persons | insert  Indicate source or key persons | insert  Indicate source or key persons |
| **Alcohol abuse** | insert  Indicate source or key persons | insert  Indicate source or key persons | insert  Indicate source or key persons | insert  Indicate source or key persons |
| **Domestic violence and abuse** | insert  Indicate source or key persons | insert  Indicate source or key persons | insert  Indicate source or key persons | insert  Indicate source or key persons |
| **Child maltreatment** | insert  Indicate source or key persons | insert  Indicate source or key persons | insert  Indicate source or key persons | insert  Indicate source or key persons |
| **Elder abuse** | insert  Indicate source or key persons | insert  Indicate source or key persons | insert  Indicate source or key persons | insert  Indicate source or key persons |
| **Racism and discrimination** | insert  Indicate source or key persons | insert  Indicate source or key persons | insert  Indicate source or key persons | insert  Indicate source or key persons |
| **Social isolation** | insert  Indicate source or key persons | insert  Indicate source or key persons | insert  Indicate source or key persons | insert  Indicate source or key persons |
| **Disadvantage and vulnerable groups (e.g. LGBT)** | insert  Indicate source or key persons | insert  Indicate source or key persons | insert  Indicate source or key persons | insert  Indicate source or key persons |
| **Gambling** | insert  Indicate source or key persons | insert  Indicate source or key persons | insert  Indicate source or key persons | insert  Indicate source or key persons |
| ***Other (when applicable: social media, bullying, etc.)*** | insert  Indicate source or key persons | insert  Indicate source or key persons | insert  Indicate source or key persons | insert  Indicate source or key persons |

## Media coverage

Highly publicized deaths by suicide often have a negative impact on suicidal ideations of vulnerable populations. Some people may identify with the person who died and come to use the same method when trying to kill oneself. It takes research efforts to determine whether or not there may be a suicide contagion, but media coverage is surely a risk factor for any vulnerable person with suicidal thoughts. It is therefore important to document if and how some suicides were reported by the media **for the past ten years** in your community. If it is not possible to obtain information for the past 10 years, we should try for **at least 3-5 years** before the start of the Roots of Hope program activities.

| **Suicide reported** | **When? Specify duration.** | **Which media(s)?** | **Frequency** | **Content. Include examples and links to online content.** | **Was the method of suicide reported? How?** |
| --- | --- | --- | --- | --- | --- |
| insert  Indicate source | insert  Indicate source | insert  Indicate source | insert  Indicate source | insert  Indicate source | insert  Indicate source |
| insert  Indicate source | insert  Indicate source | insert  Indicate source | insert  Indicate source | insert  Indicate source | insert  Indicate source |
| insert  Indicate source | insert  Indicate source | insert  Indicate source | insert  Indicate source | insert  Indicate source | insert  Indicate source |
| insert  Indicate source | insert  Indicate source | insert  Indicate source | insert  Indicate source | insert  Indicate source | insert  Indicate source |

## Public awareness campaigns and gatekeeper training

Lack of public information and stigmatization of persons with mental health problems have been shown to be major barriers to care and help seeking behaviour. Therefore, it is important to document any past or current public awareness campaigns in your community. These may include short media campaigns, long-term program, or gatekeeper training or some combination of these components.

**Important: Add additional pages to describe when more than one activity occurred.**

| **Name** | **Short media campaigns** | **Long-term programs -activities** | **Gatekeeper training** |
| --- | --- | --- | --- |
| Insert name  Insert year | insert | insert | insert |
| **Specify duration and dates of activities** | insert | insert | insert |
| **Number of participants (and number of participants trained as gatekeepers)** | insert | insert | insert |
| **Specify the objectives** | insert | insert | insert |
| **What were the means of communication?** | insert | insert | insert |
| **Describe the extent of the coverage.** | insert | insert | insert |
| **Describe resources, financial and personnel** | insert | insert | insert |
| **Who were the stakeholders?** | insert | insert | insert |
| **Evaluation undertaken? Specify the tools that had been used.** | insert | insert | insert |
| **Measures reflecting effectiveness?** | insert | insert | insert |
| **Measures reflecting cost-effectiveness?** | insert | insert | insert |
| ***Indicate source of information*** | insert | insert | insert |

## Existing mental health and suicide prevention resources

The following information aims to provide a meaningful portrait of the resources in your community. Relevant agencies include both public health institutions, such as hospitals and clinics as well as community organizations relevant to suicide prevention efforts such as helplines and crisis centres. Some agencies keep good records of their staff and users. This will help to identify priorities or gaps in particular areas of possible actions.

| **Area of focus** | | | | | |
| --- | --- | --- | --- | --- | --- |
| **Name of the organization** | **Promotion**  universal prevention (awareness programs) | **Prevention**  any service targeting those who show suicide risk factors, either directly or through relatives and friends | **Intervention**  any service targeting directly those with suicidal thoughts | **Postvention for the bereaved**  any service for people bereaved by the suicide of a loved one | **Postvention for organizations**  Workplace support after a suicide or suicide attempt |
| insert name | insert | insert | insert | insert | insert |
| **Years of service** | insert | insert | insert | insert | insert |
| **Geographic area served** | insert | insert | insert | insert | insert |
| **Vulnerable population served** | insert | insert | insert | insert | insert |
| **Numbers of staff trained in suicide prevention** | insert | insert | insert | insert | insert |
| **Identify who is trained** | insert | insert | insert | insert | insert |
| **What are the services, what technologies are being used?** | insert | insert | insert | insert | insert |
| **Hours of accessible service** | insert | insert | insert | insert | insert |
| **Number of clients served** | insert | insert | insert | insert | insert |
| **Language(s) or provision of service** | insert | insert | insert | insert | insert |
| **Measures reflecting cost-effectiveness** | insert | insert | insert | insert | insert |
| ***Sources of data*** | insert | insert | insert | insert | insert |

## Training programs for service providers

Are training programs in the area of mental health and suicide prevention for service providers available in your community? Examples include the Mental Health First Aid (MHFA) or any program from LivingWorks, e.g. the Applied Suicide Intervention Training (ASIST).

- Add additional tables for more programs

| **Name of the program** | **Specify the objectives** | **Duration, dates of activities and content** | **Who provided the training? Numbers of trainers.** | **Who was trained?** | **How many have been trained? Numbers of people trained (total and at each training).** | **What proportion of the potential service providers was trained?** |
| --- | --- | --- | --- | --- | --- | --- |
| Insert name | insert | insert | insert | insert | insert | insert |
| ***Sources of information*** | insert | | | | | |
| **Any measures of impacts or effectiveness?** | insert | | | | | |
| ***Sources of data*** | insert | | | | | |
| **Any measures of cost-effectiveness?** | insert | | | | | |
| ***Sources of data*** | insert | | | | | |

## Narrative description of your community

Please provide a two-page narrative description of your community. Describe the problems your community is facing in a detailed manner as well as community strengths and resources. Give an account of facilitators capable of responding to the identified challenges. Briefly summarize present and past activities in the area of mental health and suicide prevention. Include a historical background whenever needed as well as examples to help illustrate and substantiate the specific challenges and responses.

insert

## References

Health Nexus Santé (2015). *Six strategic steps for conducting a situational assessment*. Retrieved from <http://en.healthnexus.ca/news/six-strategic-steps-conducting-situational-assessment>

Ontario Centre of Excellence for Child and Youth Mental Health (2013). *Evidence In-Sight: Conducting a community needs assessment.* Retrieved from http://www.excellenceforchildandyouth.ca/resource-hub/conducting-community-needs-assessment

Rossi, P. H., Lipsey, M. W., & Freeman, H. E. (2004). *Evaluation: A systematic approach*. Thousand Oaks, CA: Sage.

Work Group for Community Health and Development, University of Kansas. (2013). *Chapter 3: Assessing Community Needs and Resources. The Community Tool Box*. Retrieved from <http://ctb.ku.edu/en/tablecontents/index.aspx>.

World Health Organization (2014a). *Preventing suicide: A global imperative*. Geneva, Switzerland: World Health Organization.

World Health Organization (2012). *Situation analysis and priority setting*. Retrieved from http://www.who.int/nationalpolicies/processes/priorities/en/

# **Covid-19 Situational Analysis Template**


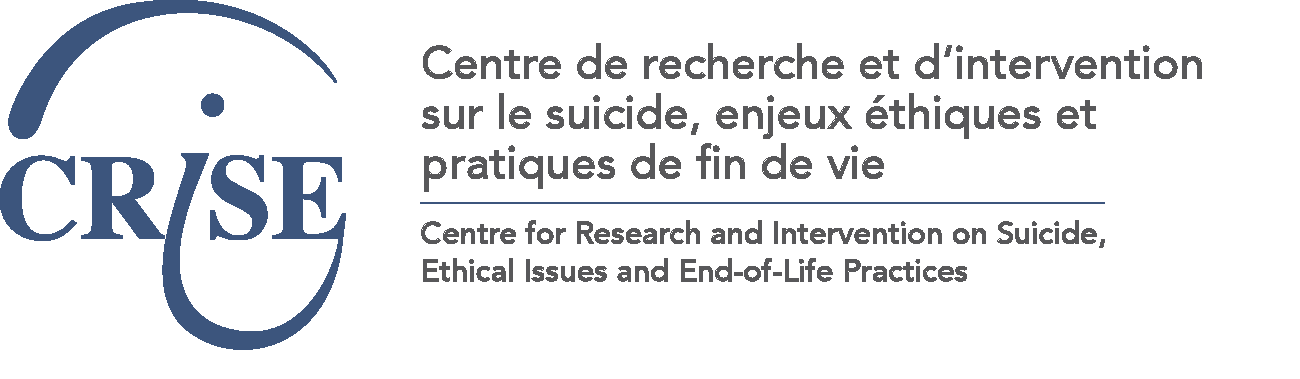


**Roots of Hope COVID-19 Situational Analysis Template for Data Collection**

**Brian L. Mishara**, Ph. D.

Principal Investigator, Evaluation of Roots of Hope

Director, Centre for Research and Intervention on Suicide, Ethical Issues and End of Life Practices (CRISE), Professor, Psychology Department, Université du Québec à Montréal 
[mishara.brian@uqam.ca](mailto:mishara.brian@uqam.ca)

**Anh Tu Tran,** Research Project Coordinator, Roots of Hope Evaluation Project, CRISE-UQAM 
[tran.anh_tu@uqam.ca](mailto:tran.anh_tu@uqam.ca)

## COVID-19 Situational Analysis for Roots of Hope Overview

This situational analysis concerning COVID-19 is a systematic process of gathering, analyzing, synthesizing and communicating available data about changes in risk and protective factors related to suicide during the Covid 19 pandemic, as well as changes in activities and services provided as part of the Roots of Hope project due to the pandemic situation. Its main objective is to support the evaluation process in assessing not only the extent to which activities are being carried out as planned, but also how they are being adapted to the new realities, and the impact of these adaptations on the population.  It should include information about:

- Changes in social determinants of health and health needs (context)
- Changes in the healthcare system and community resources (human, physical, financial, informational)
- Changes and adaptations to RoH activities
- Changes and adaptation in evaluation activities

This systematic analysis can help determine the scope of the impact of COVID-19, challenges and potential opportunities. It can help determine priorities in planning by identifying areas of possible impact (e.g. moving online), and identify crucial variables to be examined when scaling-up existing programs.

**Sources of data**

It is important to clearly indicate your sources of data.

## Contextual factors and their impacts on target populations

COVID-19 may affect your target population(s) in many ways.  Below is a list of contextual factors that should be worth documenting. You may observe changes in your community that have consequences for existing risk and protective factors. Please provide a description of the changes, indicate whether they may be protective or may increase suicide risk for your target population, and whenever possible, include quantitative data and indicate your data source.

| **Target population(s):** insert | | |
| --- | --- | --- |
| **Areas of impact** | **What may increase suicide risk** | **What may be protective** |
| **Financial or work status change** | *Examples: income loss, uncertainty about job status, etc.* | *Examples: Response benefits, bonus pay, etc.* |
|  | insert  Indicate source or key persons | insert  Indicate source or key persons |
| **Lockdown (stay-at-home) and social distancing measures** | *Examples: social isolation, psychological effects of self-isolation, domestic stress, abuse or violence, lack of social support from school or work settings, work-from-home challenges, etc.* | *Examples: social support at home, less exposure to stressors at work or school, school, less access to drugs, etc.* |
|  | insert  Indicate source or key persons | insert  Indicate source or key persons |
| **Access to mental-health services** | *Examples: people avoid seeking help because of virus fears, mental health perceived as less important, reallocation of resources, barriers to internet access, closure of resources (e.g. day centres, clinics) etc.* | *Examples:  teleconsultations may be more flexible (online or by phone), increased funding for mental health services in some provinces and jurisdictions, etc.* |
|  | insert  Indicate source or key persons | insert  Indicate source or key persons |
| **Substance use** | *Examples: substance abuse easier to hide at home, self-medication with substances to deal with isolation or stress* | *Examples: for youth fewer occasions for substance abuse and less access to drugs while at home, less money to buy drugs, less contact with “bad” influences* |
|  | insert  Indicate source or key persons | insert  Indicate source or key persons |
| **Firearms** | *Examples: increase in firearms sales because of fear, less work resulting in more hunting and risk of accidents in aboriginal communities* | *Examples: applications for firearms licenses on hold because of COVID,* |
|  | insert  Indicate source or key persons | insert  Indicate source or key persons |
| **Community response** | *Examples: lack of clarity from political leaders, lack of social distancing measure compliance from the population, etc.* | *Examples: community solidarity, more social contacts with virtual tools, etc.* |
|  | insert  Indicate source or key persons | insert  Indicate source or key persons |
| ***Other*** | insert  Indicate source or key persons | insert  Indicate source or key persons |

Indicators of suicide and suicide attempts

The COVID-19 situation may or may not increase suicide and suicide attempts in your community. Because of the very low frequency of suicidal deaths, it probably will not be possible to identify significant changes in mortality in such a limited period. However, since anxiety and isolation increase during a pandemic, it is important to document all indications sign of suicidal crises in your community, as well as significant decreases in suicidal behaviours, when they occur. Below is a proposal for some ways you could record this information. Please include all relevant **reports, links, and sources**.

|  | **Indications of increased or decreased suicidality** | **Who seems to be the population affected?** | **What are the possible relationships with COVID situation?** |
| --- | --- | --- | --- |
| **Media reports** | insert  Indicate source or key persons | insert  Indicate source or key persons | insert  Indicate source or key persons |
| **Coroner reports** | insert  Indicate source or key persons | insert  Indicate source or key persons | insert  Indicate source or key persons |
| **Hospitalisations data on suicide attempts** | insert  Indicate source or key persons | insert  Indicate source or key persons | insert  Indicate source or key persons |
| **Helplines administrative data: number of calls/ chats, number of active rescues, number and % of suicide calls, etc.** | insert  Indicate source or key persons | insert  Indicate source or key persons | insert  Indicate source or key persons |
| ***Other*** | insert  Indicate source or key persons | insert  Indicate source or key persons | insert  Indicate source or key persons |

## Planning of activities and change in infrastructure and service delivery

The COVID-19 situation has required important changes in the planning of activities: cancellations of in-person gatherings, implementation of distance-based activities (by telephone or virtual), changes to the healthcare system that may affect services (staffing, availability of mental health services), etc. Please provide a description of the changes that you have observed for each activity under each pillar, and whenever possible, include quantitative data. It is also important that you document if and how core components of the programmes had to be adapted to Covid-19 related policies and practices, such as social distancing.

|  | **Training** | **Awareness** | **Specialized supports** | **Means restriction** |
| --- | --- | --- | --- | --- |
| **Cancellation(s) of activities**  **or change in timelines** | insert  Indicate source or key persons | insert  Indicate source or key persons | insert  Indicate source or key persons | insert  Indicate source or key persons |
| **Changes in infrastructure, healthcare system (staffing, services, etc.)** | insert  Indicate source or key persons | insert  Indicate source or key persons | insert  Indicate source or key persons | insert  Indicate source or key persons |
| **Distance-based adaptations**  **(internet, text, chat or telephone)** | insert  Indicate source or key persons | insert  Indicate source or key persons | insert  Indicate source or key persons | insert  Indicate source or key persons |
| **Change(s) in core components of intended activity** | insert  Indicate source or key persons | insert  Indicate source or key persons | insert  Indicate source or key persons | insert  Indicate source or key persons |
| ***Other*** | insert  Indicate source or key persons | insert  Indicate source or key persons | insert  Indicate source or key persons | insert  Indicate source or key persons |

Measuring tools

This section emphasizes on the adaptations that were made to the measuring tools in the context of social distancing measures. Some changes are minor - e.g., moving hard-copy questionnaires online – but others may pose interpretation challenges – e.g., assessing infographics uptake with analytics. Please provide whenever applicable a description of the changes to each of the measuring tool previously submitted (surveys/questionnaires/interviews/focus groups, etc.).

| *Please add as many lines as needed for each tool* | **Indicate for which activity and under which pillar** | **Provide description of changes to the tool (data collection procedure, participants, wording, etc.)** | **Clarify changes to timelines and projected analyses** |
| --- | --- | --- | --- |
| **Tool 1 Name ___** insert | insert | insert | insert |
| **Tool 2 Name ___** insert | insert | insert | insert |
| insert | insert | insert | insert |
| insert | insert | insert | insert |
