# Supplementary material for: Evaluation of a Community Suicide Prevention Project (Roots of Hope): Protocol for an Implementation Science Study
Source: JMIR Res Protoc. 2023 Jun 14;12:e39978. doi: 10.2196/39978 (PMC10337351; doi:10.2196/39978)
Supplement: Multimedia Appendix 3 [file resprot_v12i1e39978_app3.docx]

**Multimedia Appendix 3.** Assessing planning: factors affecting planning components, methodologies, and sources of data.

| **Assessment of planning** | **Sources of data** | **Methodologies and Instruments** |
| --- | --- | --- |
| - Resources’ availability - Planning process - Level and nature of engagement - Cultural and political context - Planning facilitators, strengths and challenges - Integration of RoH with existing and other planned activities | - Key planning documents - Stakeholder focus groups - Qualitative descriptions from - coordinators (interviews) - Monitoring data from MHCC - Analyses of Situational Analysis Data - Interviews with key Informants and RoH personnel | - Planning Interview Guide - Stakeholder Focus Groups Guide - RoH Personnel Interview Guides - Situational Analysis Template   - Further analyses by local teams when needed   - Data to be collected as needed   - Recurring monitoring data to be identified and collected - Review of Key Documents |
