# Supplementary material for: Evaluation of a Community Suicide Prevention Project (Roots of Hope): Protocol for an Implementation Science Study
Source: JMIR Res Protoc. 2023 Jun 14;12:e39978. doi: 10.2196/39978 (PMC10337351; doi:10.2196/39978)
Supplement: Multimedia Appendix 4 [file resprot_v12i1e39978_app4.docx]

**Multimedia Appendix 4.** Assessing implementation: adequacy of services and activities to target populations and equity issues, methodologies, and sources of data.

| **Assessment of implementation** | **Sources of data** | **Methodologies and Instruments** |
| --- | --- | --- |
| - Identification of target populations | - Community focus groups & interviews with key informants and RoH personnel - Analyses of Situational Analysis data | - Community Focus Groups Template - Focus groups with population - Key Informants & RoH personnel Interview Guides, Focus Group Guides - Edmonton's instruments to be incorporated   - Stakeholder interview   - Service provider survey - Narrative interview with people impacted by suicide - Situational Analysis data on sub-groups: local analyses when needed |
