# Supplementary material for: Evaluation of a Community Suicide Prevention Project (Roots of Hope): Protocol for an Implementation Science Study
Source: JMIR Res Protoc. 2023 Jun 14;12:e39978. doi: 10.2196/39978 (PMC10337351; doi:10.2196/39978)
Supplement: Multimedia Appendix 5 [file resprot_v12i1e39978_app5.docx]

**Multimedia Appendix 5.** Training pillar: implementation common metrics, methodologies, and sources of data.

| **Assessment of implementation** | **Sources of data** | **Methodologies and Instruments** |
| --- | --- | --- |
| - Delivery of services/activities by pillar - Available - Acceptable - Accessible - High Quality - Equitable - Target populations receive activities/services as intended | - Number and proportion of trained/attendees: administrative data and Community Action Plan - Participation rate and drop-outs (attendance, website analytics): administrative data - Location and time of sessions/resources distribution, internet access: administrative data - Participants' demographics and conformity with target population characteristics: surveys - Quality assessment of sessions/resources/activities - Qualitative data from interviews with coordinators community focus groups and key informants - Target population surveys and focus groups (pre and post measures) | - Living Works questionnaires (revised from CRISE)   - Quality of training and participants' demographics - Local Coordinators, RoH personnel and Key Informants Implementation Interview Guides: analyses of qualitative assessments of activities to be compared with Action Plan - Training End-Users Surveys to assess accessibility and acceptability - Local instruments to be incorporated   - Saskatchewan: training follow-up questionnaire   - Edmonton: third party evaluations components |
