# Supplementary material for: Evaluation of a Community Suicide Prevention Project (Roots of Hope): Protocol for an Implementation Science Study
Source: JMIR Res Protoc. 2023 Jun 14;12:e39978. doi: 10.2196/39978 (PMC10337351; doi:10.2196/39978)
Supplement: Multimedia Appendix 6 [file resprot_v12i1e39978_app6.docx]

**Multimedia Appendix 6.** Awareness pillar: implementation common metrics, methodologies, and sources of data.

| **Assessment of implementation** | | **Sources of data** | | **Methodologies and Instruments** | |
| --- | --- | --- | --- | --- | --- |
| - Delivery of services/activities by pillar   - Available   - Acceptable   - Accessible   - High Quality   - Equitable - Target populations receive activities/services as intended | | - Number and proportion of attendees: administrative data and Community Action Plan - Participation rate and drop-outs (attendance, website analytics): administrative data - Location and time of events/resources distribution, internet access: administrative data - Participants' demographics and conformity with target population characteristics: surveys - Quality assessment of sessions/resources/awareness activities - Qualitative data from interviews with coordinators community focus groups and key informants - Target population surveys and focus groups (pre and post measures) | | - Awareness Community Survey to assess quality of activity and accessibility - Local Coordinators, Personnel and Key Informants Implementation Interview Guides to assess conformity - Local instruments to be incorporated - Burin Peninsula: Community Suicide Awareness Presentation Survey (stigma) - Edmonton: Campaign survey by polling firm | |
