# Supplementary material for: Evaluation of a Community Suicide Prevention Project (Roots of Hope): Protocol for an Implementation Science Study
Source: JMIR Res Protoc. 2023 Jun 14;12:e39978. doi: 10.2196/39978 (PMC10337351; doi:10.2196/39978)
Supplement: Multimedia Appendix 7 [file resprot_v12i1e39978_app7.docx]

**Multimedia Appendix 7.** Specialized support pillar: implementation common metrics, methodologies, and sources of data.

| **Assessment of implementation** | **Sources of data** | **Methodologies and Instruments** |
| --- | --- | --- |
| - Delivery of services/activities by pillar - Available - Acceptable - Accessible - High Quality - Equitable - Target populations receive activities/services as intended | - Number and proportion of those receiving services: administrative data and Community Action Plan - Participation rate and drop-outs (attendance, website analytics): administrative data - Location and time of sessions/resources distribution, internet access: administrative data - Participants' demographics and conformity with target population characteristics: surveys - Quality assessment of sessions/services - Qualitative data from interviews with coordinators community focus groups and key informants - Target population surveys and focus groups (pre and post measures) | - Specialized Support Users Survey to assess quality of services, participants' demographics, availability and accessibility - Saskatchewan: Grief Recovery Satisfaction Survey - Waterloo-Wellington: "Why" support after suicide loss questionnaire - Available, accessible   - Analyses of trends in wait times - Service Providers Survey to address acceptability - Coordinators, personnel and Key informants Interview Guides; Focus Group guides to address conformity - Analyses of quantitative data to address quantitative indicators |
