# Supplementary material for: Evaluation of a Community Suicide Prevention Project (Roots of Hope): Protocol for an Implementation Science Study
Source: JMIR Res Protoc. 2023 Jun 14;12:e39978. doi: 10.2196/39978 (PMC10337351; doi:10.2196/39978)
Supplement: Multimedia Appendix 8 [file resprot_v12i1e39978_app8.docx]

**Multimedia Appendix 8.** Means restriction pillar: implementation common metrics, methodologies, and sources of data.

| - **Assessment of implementation** | - **Sources of data** | - **Methodologies and Instruments** |
| --- | --- | --- |
| - Delivery of services/activities by pillar   - Available   - Acceptable   - Accessible   - High Quality   - Equitable - Target populations receive activities/services as intended | - Number and proportion of those receiving resources: administrative data and Community Action Plan - Participation rate and drop-outs (applicable for round-up campaign) (attendance, website analytics): administrative data - Location and time of resources distribution, internet access: administrative data - Participants' demographics and conformity with target population characteristics: surveys - Quality assessment of resources - Qualitative data from interviews with coordinators community focus groups and key informants - Target population surveys and focus groups (pre and post measures) | - Community Focus Groups Template to assess quality of resources distributed, accessibility - Coordinators, RoH personnel and Key informants Interview Guides to address conformity and acceptability |
