# Supplementary material for: Evaluation of a Community Suicide Prevention Project (Roots of Hope): Protocol for an Implementation Science Study
Source: JMIR Res Protoc. 2023 Jun 14;12:e39978. doi: 10.2196/39978 (PMC10337351; doi:10.2196/39978)
Supplement: Multimedia Appendix 9 [file resprot_v12i1e39978_app9.docx]

**Multimedia Appendix 9.** Short-term outcomes, methodologies, and sources of data.

| - **Assessment of outcomes** | | - **Sources of data** | | - **Methodologies and instruments** | |
| --- | --- | --- | --- | --- | --- |
| - Short-term and intermediate outcomes in services/practices   - Delivery capacity   - Treatment gaps and inequities   - Skills and knowledge of service providers | | - Community focus groups & interviews with key informants - Analyses of routinely collected data (Situational Analysis data, administrative data) | | - Community Focus Groups Templates - Key Informants and RoH personnel Interview Guides - Service Providers Survey and Questionnaires to assess knowledge and skill   - Living Works questionnaires - Trends analyses of administrative data | |
| - Short-term and intermediate outcomes in community - Community empowerment (perceptions and barriers to accessing help), quality of life - Attitudes (stigma), behaviors (help-seeking) - General knowledge and awareness (of services, on suicide and mental health) | | - Community surveys (pre-post measures of stigma, resilience, attitudes, knowledge and use of resources) - Community focus groups (pre-post measures) & interviews with key informants - Analyses of routinely collected data (administrative data on use of services) | | - Measure of Stigma   - Stigma of Suicide Scale (SOSS), short form - General Knowledge Assessment   - Literacy of Suicide Scale (LOSS) - Help-seeking Assessment   - General Help-Seeking Questionnaire (GHSQ) - Community focus groups Template - Analyses of media reports - Analyses of statistical and administrative data | |
