# Supplementary material for: Evaluation of a Community Suicide Prevention Project (Roots of Hope): Protocol for an Implementation Science Study
Source: JMIR Res Protoc. 2023 Jun 14;12:e39978. doi: 10.2196/39978 (PMC10337351; doi:10.2196/39978)
Supplement: Multimedia Appendix 10 [file resprot_v12i1e39978_app10.docx]

**Multimedia Appendix 10.** Impact on suicidal behaviors, quality of life, community empowerment, experience of care, and effects on practices. 

| **Assessment of Impact** | **Sources of data** | **Methodologies and instruments** |
| --- | --- | --- |
| - Suicide deaths - Suicide attempts - Lives of those affected by suicide improved | - Number and rates of suicide deaths as collected by provincial authorities/ Statistics Canada/ Coroners* - Number and rates of suicide attempts as recorded by provincial authorities, municipal and hospital data* - “Lived Experience” / those who attempted suicide or were at high risk: mental well-being and quality of life measures (pre and post measures) & Persons bereaved by Suicide (“survivors”) - Assessments (pre-post) of changes in knowledge, attitudes, behaviors (help-seeking and service use) - Surveys on stigma - Surveys on experience of care - Qualitative data from focus groups, interviews with key informants on empowerment | - Analyses of suicide deaths trends - Analyses of hospitalization trends; Statistics Canada data* - Analyses of administrative data - Help-seeking behavior survey: general population and target populations   - General Help Seeking Questionnaire (GHSQ) - Mental well-being measures: general population and target populations   - General Health Questionnaire GHQ-28 - Local specific measure to be incorporated: Edmonton’s interview with people with lived experience - Quality of life measures: general population and target populations   - The World Health Organization Quality of Life (WHOQOL) - Service providers Knowledge and Attitudes assessment   - Literacy of Suicide Scale (LOSS)   - Living Works data analyses on training metrics - Measure of Stigma: general population and target populations   - Stigma of Suicide Scale (SOSS), short form - End-users Experience of care Survey   - The Ontario Perception of Care Tool for Mental Health and Addictions (OPOC-MHA) - Practices Assessment Key Informants Interviews Guide - Community Focus Groups Template to assess empowerment |

_*aggregation may be needed when low incidence poses risk to privacy if reported individually_
