# Supplementary material for: Evaluation of a Community Suicide Prevention Project (Roots of Hope): Protocol for an Implementation Science Study
Source: JMIR Res Protoc. 2023 Jun 14;12:e39978. doi: 10.2196/39978 (PMC10337351; doi:10.2196/39978)
Supplement: Multimedia Appendix 11 [file resprot_v12i1e39978_app11.docx]

**Multimedia Appendix 11.** Summary of common measures and assessments for quantitative and qualitative data.

| **Overarching Questions through qualitative appreciation**  Model Appropriateness, Contextual Facilitators and Impediments, RoH coordination with other agencies, Resources Allocation, Impact of Covid-19, Impact of evaluation research CoP, Implication for Policies and Scaling-Up, as well as recommendations for other communities | |
| --- | --- |
| - Review of Key Documents - Review of Local Researchers' Reports - RoH Coordinators, Implementation and Research Personnel Interview Guide - Stakeholders Interview Guides and Focus Groups Templates | |
| **Roots of Hope Planning** | |
| Quantitative Assessment | Qualitative Assessment |
| - Situational Analysis Template - COVID-19 Situational Analysis Template | - Planning and Personnel Interview Guides - Stakeholder Focus Groups Template - Review of MHCC key documents (Community Action Plans) - Situational Analysis Qualitative Assessments (General and COVID-19) |
| **Roots of Hope Implementation** | |
| Target population identification : quantitative analysis | Target identification: qualitative analysis |
| - Situational Analysis Template | - Community Focus Groups Templates - Key Informants Interview Guide - Planning and Personnel Interview Guides - Review of MHCC and community key documents |
| Quantitative assessment of Conformity, Coverage, Dosage, Quality,  Acceptability and Equity of activities | Qualitative Assessment of Conformity, Acceptability and Quality of activities |
| - Administrative data - Living Works Questionnaires - End-users' surveys - Community Surveys - Service Providers Surveys | - Coordinators & Personnel Interview Guides - Key Informants Interview Guides - Community Focus Groups Templates |
| **Roots of Hope Effects** | |
| Quantitative assessment per sub-groups | Qualitative Assessment of Conformity, Acceptability and Quality of activities |
| - Analysis of statistical data and administrative data (suicide, hospitalizations, Statistics Canada) - Experience of care Survey: Ontario Perception of Care Tool for Mental Health and Addictions OPOC-MHA) - Help-Seeking Behavior: General Help-Seeking Questionnaire GHSQ - Quality of Life: The World Health Organization Quality of Life (WHOQOL) - Mental Well-Being: General Health Questionnaire GHQ-28 - Stigma: Stigma of Suicide Scale (SOSS) - General Knowledge: Literacy of Suicide Scale (LOSS) - Service Providers Knowledge and Attitudes: Living Works Assessments and LOSS | - Community Focus Groups Templates - Key Informants and RoH Personnel Interview Guides - Analysis of Media Reports |
